# The effect of blood cells retained in rat livers during static cold storage on viability outcomes during normothermic machine perfusion

**DOI:** 10.1038/s41598-021-02417-6

**Published:** 2021-11-30

**Authors:** Omar Haque, Casie A. Pendexter, Benjamin T. Wilks, Ehab O. A. Hafiz, James F. Markmann, Korkut Uygun, Heidi Yeh, Shannon N. Tessier

**Affiliations:** 1 Center for Engineering in Medicine and Surgery, Massachusetts General Hospital, 51 Blossom St, Boston, MA 02114 USA; 2 Shriners Hospitals for Children, Boston, MA USA; 3 Department of Surgery, Beth Israel Deaconess Medical Center, Boston, MA USA; 4 Department of Surgery, Division of Transplantation, Center for Transplantation Science, Massachusetts General Hospital, 32 Fruit Street, Boston, MA 02114 USA; 5 Harvard Medical School, Boston, MA USA; 6 Department of Electron Microscopy Research, Theodor Bilharz Research Institute, Giza, Egypt

**Keywords:** Hepatology, Liver

## Abstract

In transplantation, livers are transported to recipients using static cold storage (SCS), whereby livers are exposed to cold ischemic injury that contribute to post-transplant risk factors. We hypothesized that flushing organs during procurement with cold preservation solutions could influence the number of donor blood cells retained in the allograft thereby exacerbating cold ischemic injury. We present the results of rat livers that underwent 24 h SCS after being flushed with a cold University of Wisconsin (UW) solution versus room temperature (RT) lactated ringers (LR) solution. These results were compared to livers that were not flushed prior to SCS and thoroughly flushed livers without SCS. We used viability and injury metrics collected during normothermic machine perfusion (NMP) and the number of retained peripheral cells (RPCs) measured by histology to compare outcomes. Compared to the cold UW flush group, livers flushed with RT LR had lower resistance, lactate, AST, and ALT at 6 h of NMP. The number of RPCs also had significant positive correlations with resistance, lactate, and potassium levels and a negative correlation with energy charge. In conclusion, livers exposed to cold UW flush prior to SCS appear to perform worse during NMP, compared to RT LR flush.

## Introduction

In the United States, the demand for transplantable livers far outstrips supply, with over 12,000 patients on the waitlist for a liver transplant but less than 9000 transplants done annually^1,2^. One challenge in liver transplantation that contributes to this organ shortage is damage to the liver graft during transportation using static cold storage (SCS). Cold ischemic time (CIT) of a liver graft is defined as the interval between in situ cold preservation flush of the organ during procurement to removal of the graft from 4 °C SCS at time of implantation^3^. Cold ischemia is a known driver of ischemia reperfusion injury as the liver transitions from oxygen limiting conditions at 4 °C to higher metabolic rates at normothermic temperatures during implantation. Longer CITs have been shown to lead to higher rates of early allograft dysfunction^4^ and primary nonfunction^5^. Despite the success of SCS in organ transportation, more work is required to fully uncover the mechanisms of cold ischemic injury on the liver, and the impact of methods that prepare the liver allograft for SCS remain understudied.

During clinical organ procurement, the donor liver is perfused in situ through the aorta and portal vein (PV) using cold (4 °C) University of Wisconsin (UW) solution. Once the donor hepatectomy is complete, retained blood in the liver is flushed out using additional UW solution on the back table until clear fluid emerges from the inferior vena cava (IVC)^6^. The temperature, composition, and viscosity of flushing solution has been shown to be an important determination of liver graft function after transplant^7,8^. However, even after cold flushing, donor blood remains in the liver allograft, which may be harmful to resident cells, especially during SCS.

Platelets, neutrophils, and red blood cells (RBCs) are well known to be affected by thermal stresses. For example, hypothermic conditions promote platelet activation and aggregation by changing platelet shape, increasing expression of adhesion molecules, and promoting margination^9,10^. Neutrophils also respond to physiologic stressors by releasing DNA into the extracellular space^11,12^. This process called NETosis leads to microvasculature vaso-occlusions^13^, and has been shown to be exacerbated by cold exposure^14,15^. Finally, RBCs are more resistant to hydrodynamic dispersion with decreasing temperature, promoting aggregation^16^ and rouleaux formation^17^. Hypothermia has also been linked to coagulopathy due to decreased fibrinogen availability^18^.

In addition to the direct impact of temperature on blood cells, cold temperatures may also adversely affect liver transplant flushing solutions. Post et al. showed that flushing with warm lactated ringers (LR) resulted in less RBC retention and shorter washout times compared to cold UW and histidine tryptophan ketoglutarate (HTK) flushes^19^. Additional studies demonstrated that the high potassium concentration in UW solution combined with cold temperature resulted in vasoconstriction^20^, and the high viscosity of UW lead to poor initial reperfusion of the liver graft due to RBC aggregation^21^.

Prior literature has shown that the interacting factors of cold temperature, solution composition, and viscosity of flushing solutions interact to influence donor blood cell retention/aggregation as well as allograft physiology. Based on this knowledge, we hypothesized that livers procured with a room temperature (RT) lactated ringers (LR) flush would perform better than a cold flush with UW and lead to less retained peripheral cells (RPCs) from inadequate liver graft flushing. To accomplish these aims, we used an *ex-vivo* rat liver normothermic machine perfusion model^22–26^ to study differences in hepatic function after a variety of flush and SCS conditions.

## Materials and methods

### Experimental design

**Figure 1:**
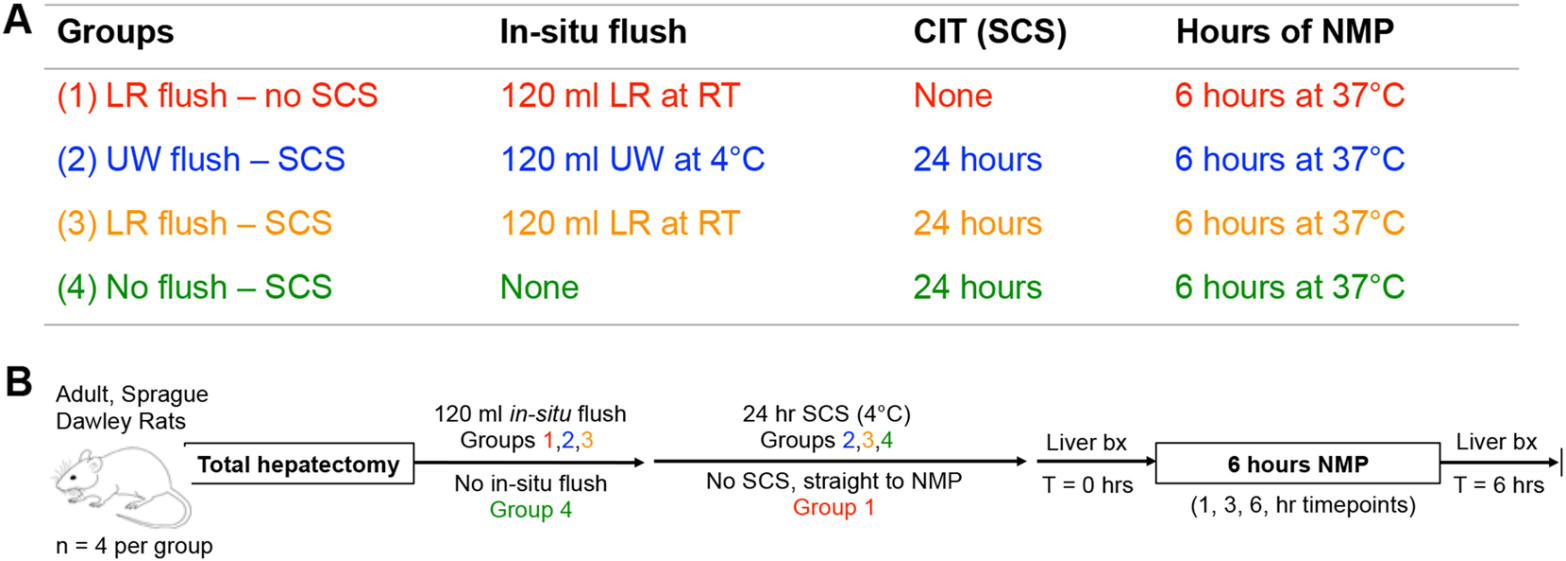
Experimental design. (**A**) Flush group designation (n = 4) and (**B**) experimental design. Each group of adult, Sprague Dawley rats underwent total hepatectomy with an in-situ flush (except group 4 which had no flush), following by 24 h of SCS (except group 1 which went straight to NMP). Liver biopsies for histology were taken prior to perfusion and after 6 h of perfusion, and perfusion metrics were recorded at 1, 3, and 6 h timepoints.

The study is reported in accordance with ARRIVE guidelines. The livers of adult Sprague Dawley rats were procured and subjected to one of the following conditions (n = 4 per group): (1) RT LR flush with no SCS, (2) cold UW flush with 24 h SCS, (3) RT LR flush with 24 h SCS, or (4) no flush with 24 h SCS (Fig. 1A). Prior work has shown that rat livers have synthetic function on ex-vivo machine perfusion and 100% transplant survival after 24 h of SCS^23^. Hence 24 h SCS represents our control group that mirrors ischemia times of ~ 9–12 h in human livers^27^.

After these flush and SCS conditions were completed, the livers were subjected to 6 h of NMP. Allocation of livers was arbitrarily alternated between the four experimental groups. Perfusion metrics, injury markers, weight gain, ATP, and histology were compared between the four groups. Figure 1B summarizes the experimental design.

### Liver procurement

Livers from healthy, adult Sprague Dawley rats (12 weeks old, weighing 250–400 g) (Charles River Laboratories, Wilmington, MA, USA) were used for all experiments to ensure comparable baseline characteristics between the 4 groups. The animals were stored in a humidity and temperature-controlled room with a natural light/dark cycle, socially housed (according to weight standards) with bedding, and were provided unrestricted access to food and water in accordance with the National Research Council guidelines. The health of the rats was maintained by Massachusetts General Hospital (MGH) Center of Comparative Medicine (CCM), and all experimental protocols were approved by the Institutional Animal Care and Use Committee (IACUC Protocol Number 2016N000564) of MGH.

Donor Sprague Dawley rats were anesthetized by inhalation of isoflurane. The animal was placed on a surgical table in supine position. The abdomen was opened with a transverse abdominal incision with scissors. Ligaments and adhesions at the superior and posterior aspect of the liver were divided and the liver was freed from its surroundings. Heparin (1 U/g rat) was injected into the intrahepatic inferior vena cava (IVC). The hepatic artery was dissected and ligated and the bile duct (BD) was cannulated with a 28G catheter and secured in place with 6-0 silk suture. The gastric and splenic vein branches of the portal vein (PV) were ligated, and the PV was cannulated with a 16G angiocatheter and secured with 4-0 silk suture. The infrahepatic IVC was transected immediately upon cannulation of the portal to vent flush outflow flush and to euthanize the rat by exsanguination.

### Liver flush and static cold storage

Rat livers were flushed and stored based on the group designation. Group 1 livers were flushed with 120 mL of room temperature (RT) lactated ringers (LR) and subsequently were connected to a normothermic perfusion pump. Group 2 livers were flushed with 120 mL of 4 °C UW (Bridge to Life, Columbia, SC, USA) and then underwent 24 h of SCS at 4 °C. Group 3 livers were flushed with 120 mL of RT LR followed by 24 h of SCS at 4 °C. The livers were flushed at a rate of 15–20 mL/min for a total flush duration of 6–8 min for a 120 mL flush. Also, following the initial flush, livers that were going to be cold stored were also flushed with 5 mL of UW solution at 1 mL/min for a total flush duration of 5 min to mimic human liver cold storage conditions. Finally, group 4 livers had no in-situ flush followed by 24 h of SCS at 4 °C. Upon completion of the flush (except group 4), the liver was freed from its remaining attachments to the diaphragm, retroperitoneum, and intestine, and the suprahepatic IVC was transected and the liver was removed from the body. For SCS, the rat livers were placed in a storage bag with 50 mL of UW solution at 4 °C for 24 h.

### Normothermic machine perfusion

Following SCS for 24 h (except Group 1), all rat livers were placed on NMP at 37 °C for 6 h to assess liver viability. Ex-vivo NMP with Sprague Dawley rats was performed with a sterilized perfusate consisting of 500 mL of William’s E media (Sigma-Aldrich, St. Louis, MO, USA), 20 g of bovine serum albumin (Sigma-Aldrich), 20 g of polyethylene glycol 35,000 (PEG) (Sigma-Aldrich), 20 mg of dexamethasone (Sigma-Aldrich), 2 mL of heparin (MGH pharmacy), 1 mL of regular insulin (MGH pharmacy), 10 mL penicillin–streptomycin (Thermo Fisher Scientific, Waltham, MA, USA), and 2.2 g of sodium bicarbonate (Hospira, Lake Forest, Il, USA) to obtain a pH of 7.4. The perfusate was acellular so any retained peripheral cells quantified on histology after perfusion were from the liver procurement and not reflective of any cellular deposition from a cell based perfusate. Since NMP typically uses red blood cells mixed in the perfusate, the perfusion-based viability metrics presented herein should be interpreted accordingly. The perfusate was circulated at 37 °C at 30 mL/min through an oxygenator with 95% O_2_ and 5% CO_2_ at 1 L/min to achieve a maximum partial oxygen pressure of > 500 mmHg and un-depleted oxygen outflow > 150 mmHg to ensure adequate liver graft oxygenation. The PV intrahepatic inflow pressure was kept under 11 mmHg.

Perfusate entered the PV and exited freely from the supra-hepatic vena cava (SHVC) and IVC. The perfusion circuit consisted of a perfusion chamber, two Masterflex peristaltic pumps (Cole Parmer, Vernon Hill, IL), a membrane oxygenator (Radnoti, Monrovia, CA, USA), a heat exchanger (Radnoti), and a bubble trap (Radnoti). Liver temperature was regulated with a water bath (Lauda, Brinkmann, Westbury, NY, USA) and constantly monitored. During the 6-h perfusion, perfusion pH was maintained at 7.35–7.45 with the supplementation of sodium bicarbonate as necessary^25^.

### Perfusion assessment

Two sampling ports were placed upstream of the PV and in the perfusion chamber to collect lab values for rat liver perfusion performance (resistance, lactate, oxygen consumption, and weight change) and assessment of injury (AST, ALT, potassium). Perfusate samples were collected at 1, 3, and 6 h. Chemistry and blood gases were analyzed on an i-STAT blood Analyzer (Abbott Point of Care Inc., Princeton, NJ, USA) and liver function tests (LFTs) were analyzed on a Piccolo Xpress (Abaxis, Union City, CA, USA). Resistance was defined as pressure divided by flow rate, adjusted for liver weight in grams (g), and oxygen consumption was defined as inflow pO_2_ minus outflow pO_2_, adjusted for flow rate and liver weight. Weight change (%) was defined as the change in liver weight before and after perfusion, divided by the weight before perfusion.

Analysis of ATP and energy charge (EC) was conducted with additional (~ 1 g) tissue biopsies taken before and after NMP. These 3 mm wedge liver biopsies were taken at the periphery of the left lateral lobe with Metzenbaum scissors. These samples were flash frozen in liquid nitrogen and stored at − 80 °C. Measurement of ATP and EC was performed using a liquid chromatography-mass spectrometry (LC–MS) at the Shriners Hospital-Boston Mass Spectrometry Core Facility (Boston, MA, USA). Energy charge was defined as [(ATP × 0.5 ADP)/(ATP + ADP + AMP)].

### Histological analysis

Liver biopsies were acquired before perfusion and after 6 h of NMP. Rat liver tissue was fixed in 10% formalin for 24 h, stored in 70% (v/v) ethanol, embedded in paraffin, sectioned, and stained with Hematoxylin and Eosin (H&E), Terminal deoxynucleotidyl transferase dUTP nick end labeling (TUNEL), Periodic acid-Schiff (PAS)-diastase (Sigma-Aldrich), and Reticulin (Polysciences, Warrington, PA, USA) to assess cellular architecture and quantify the number of RPCs. Hematoxylin stains nuclei blue and eosin stains the extracellular matrix and cytoplasm pink. TUNEL labels the 3′-hydroxyl termini in double stranded DNA breaks that are generated during apoptosis and was the primary stain in this study to qualitatively evaluate for DNA damage over 20 high power fields (HPFs)^28^. PAS stains polysaccharides such as glycogen magenta and is used in combination with diastase which breaks down glycogen (to differentiate glycogen from other PAS positive elements)^29^. Finally, Reticulin is a silver stain that is used to visualize reticular fibers in liver histopathology^30^. All tissue processing was performed at the MGH Histology Molecular Pathology Core Facility (Boston, MA, USA) and analyzed by a blinded pathologist (E.O.A.H.).

### Statistical analysis

Statistical analysis was performed with Prism 8 software Version 9.1.2 (GraphPad Software, San Diego, CA, USA, graphpad.com) with a two-sided significance level of 0.05. Repeated measures of two-way Analysis of variance (ANOVA) were used for the comparison of the time-course perfusion data, followed by Tukey’s post-hoc test to examine statistically significant differences between the four flush groups and to correct for multiple comparisons. Perfusion metrics were reported as means with standard deviations as error bars. Strength of association between resistance, lactate, potassium, and EC, and RPCs per 20 HPFs were assessed with Pearson correlation coefficients (r).

## Results

### Perfusion metrics

**Figure 2:**
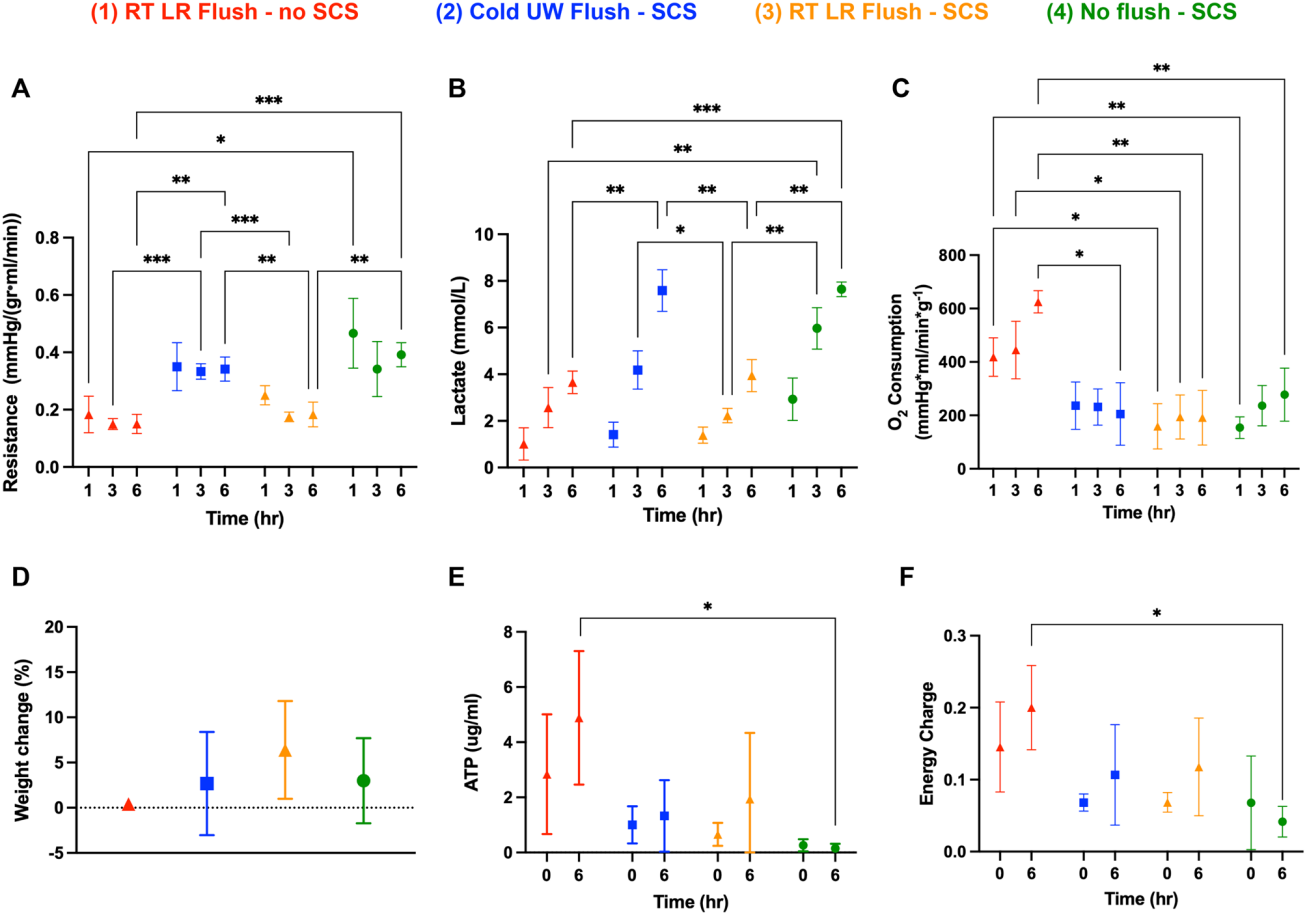
Perfusion metrics of 6 h ex-vivo rat liver NMP detailing (**A**) R, intrahepatic resistance, (**B**) lactate, (**C**) oxygen consumption, (**D**) percent liver weight change, (**E**) ATP, and (**F**) energy charge. RT LR flush livers with no SCS (red, n = 4) had the lowest resistance, the lowest lactate levels, and the highest oxygen consumption while no flush livers with SCS (green, n = 4) had the highest resistance and lactate levels. Compared to the cold UW flush livers with SCS (blue, n = 4), RT LR flush livers with SCS (orange, n = 4) had significantly higher lower resistance and lactate levels at 6 h of NMP. RT LR flush livers (red) had significantly higher ATP and EC compared to no flush livers (green) at 6 h of NMP. Stars denote statistical significance (ANOVA, followed by Tukey’s post-hoc test): *0.01 < p < 0.05; **0.001 < p < 0.01; ***0.0001 < p 0.001. Icons represent means and error bars represent standard deviations.

Resistance, lactate, oxygen consumption, weight change, ATP, and EC were monitored throughout the 6 h perfusion to assess liver performance. RT LR flush livers with no SCS (group 1) had lowest mean intrahepatic resistance on NMP at 1 h [0.18 ± 0.064 mmHg/(g mL/min)], 3 h [0.15 ± 0.019 mmHg/(g mL/min)], and 6 h [0.15 ± 0.033 mmHg/(g mL/min)], while no flush livers with SCS (group 4) had the highest mean intrahepatic resistance on NMP at 1 h (0.47 ± 0.12 mmHg/(g mL/min)], 3 h [0.34 ± 0.096 mmHg/(g mL/min)], and 6 h [0.39 ± 0.042 mmHg/(g mL/min)]. Compared to the clinical standard of cold UW flush livers with SCS (group 2), RT LR flush livers with SCS (group 3) had lower mean intrahepatic resistance at 3 h (0.33 ± 0.027 vs 0.17 ± 0.017 mmHg/(g mL/min), p = 0.0007) and 6 h (0.34 ± 0.042 vs 0.18 ± 0.043 mmHg/(g mL/min), p = 0.0075) (Fig. 2A).

RT LR flush livers with no SCS (group 1) had the lowest mean lactate levels on NMP at 1 h (1.01 ± 0.69 mmol/L) and 6 h (3.6 ± 0.48 mmol/L) while no flush livers SCS (group 4) had the highest mean lactate levels at 1 h (2.9 ± 0.91 mmol/L), 3 h (6.0 ± 0.89 mmol/L), and 6 h (7.6 ± 0.31 mmol/L). Compared to the clinical standard of cold UW flush livers with SCS (group 2), RT LR flush livers with SCS (group 3) had significantly lower mean lactate levels at 3 h (4.2 ± 0.82 vs 2.2 ± 0.3 mmol/L, p = 0.04) and 6 h (7.6 ± 0.89 vs 3.9 ± 0.69 mmol/L, p = 0.003) (Fig. 2B).

RT LR flush livers with no SCS (group 1) had the highest mean level of oxygen consumption on NMP at 1 h (278 ± 48 mmHg), 3 h (297 ± 72 mmHg), and 6 h (417 ± 28 mmHg). By 6 h of NMP, RT LR flush livers with no SCS had a higher mean level of oxygen consumption (417 ± 28 mmHg) compared to group 2 livers (137 ± 78 mmHg, p = 0.01), group 3 livers (127 ± 68 mmHg, p = 0.005), and group 4 livers (185 ± 66, p = 0.01). There were no statistically significant differences in mean oxygen consumption between groups 2, 3, and 4 (Fig. 2C).

RT LR flush livers with no SCS (group 1) had the lowest mean percent weight increase (0.40 ± 0.58%) while RT LR flush livers with SCS (group 3) had the highest (6.4 ± 5.4%). There were no statistically significant differences in mean percent weight change before and after NMP between the 4 groups (Fig. 2D).

ATP and EC were calculated at T = 0 (before perfusion) and T = 6 h (after perfusion). RT LR flush livers with no SCS (group 1) had the highest mean ATP levels at T = 0 (2.8 ± 2.17 μg/mL) while no flush livers with SCS (group 4) had the lowest mean ATP levels at T = 0 (0.26 ± 0.22 μg/mL). There were no statistically significant differences between the 4 groups before perfusion. At T = 6 h, group 1 livers continued to have the highest mean ATP levels (4.9 ± 2.4 μg/mL) while group 4 livers continued to have the lowest levels (0.15 ± 0.16 μg/mL). There were no statistically significant differences between group 2 and 3 livers (Fig. 2E).

Similar to ATP levels, at T = 0 group 1 livers had the highest mean EC (0.15 ± 0.062) while group 4 livers had the lowest (0.68 ± 0.065). There were no statistically significant differences between the 4 groups before perfusion. At T = 6 h, group 1 livers continued to have the highest mean EC (0.20 ± 0.058) while group 4 livers continued to have the lowest (0.042 ± 0.021). There were no statistically significant differences between group 2 and 3 livers (Fig. 2F).

### Perfusion injury markers

**Figure 3:**
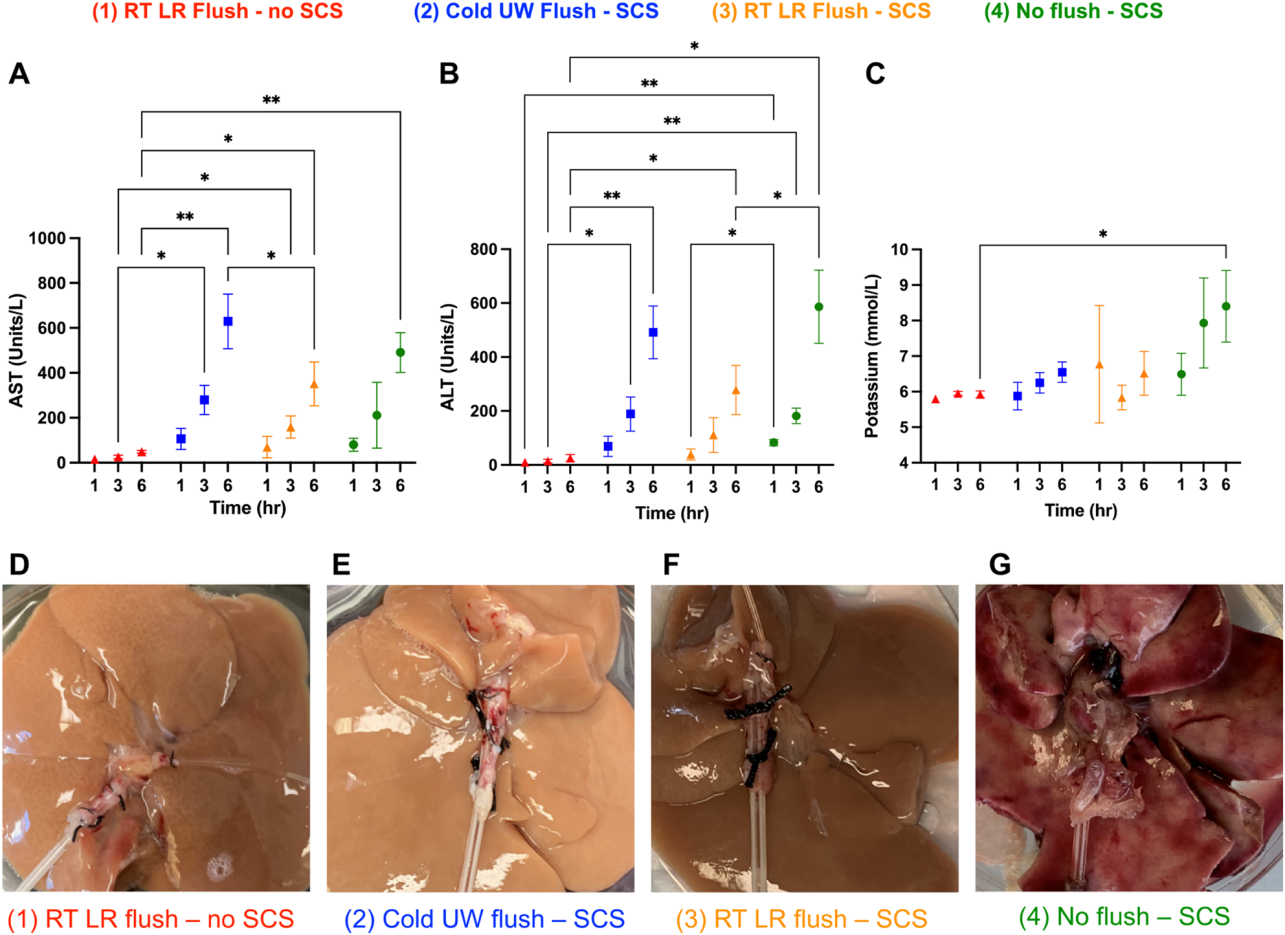
Perfusion injury markers of 6 h ex-vivo rat liver NMP detailing (**A**) AST, (**B**) ALT, (**C**) perfusate potassium, and (**D**–**G**) gross liver appearance after perfusion. RT LR flush livers with no SCS (red, n = 4) and RT LR flush livers with SCS (orange, n = 4) met transplantable criteria for AST and ALT levels while cold UW flush livers with SCS (blue, n = 4) and no flush livers with SCS (green, n = 4) did not. Grossly, group 1 and 3 livers appeared normal after perfusion while group 2 livers appeared pale from cellular washout and group 4 livers had major areas of infarction and necrosis. Stars denote statistical significance (ANOVA, followed by Tukey’s post-hoc test): *0.01 < p < 0.05; **0.001 < p < 0.01. Icons represent means and error bars represent standard deviations.

AST, ALT, and perfusate potassium levels were recorded to assess the degree of hepatic injury during perfusion. RT LR flush livers with no SCS (group 1) had the lowest mean AST levels on NMP at 1 h (16 ± 5.0 units/L), 3 h (25 ± 8.1 units/L), and 6 h (48 ± 5.7 units/L) while cold UW flush livers with SCS (group 2) actually had the highest mean AST levels at 1 h (106 ± 47 units/L), 3 h (279 ± 65 units/L), and 6 h (629 ± 121 units/L). Both group 1 and group 3 livers had mean AST levels within transplantable criteria during 6 h of NMP while group 2 and group 4 livers did not meet AST transplantable criteria by 6 h^25,31^. Compared to cold UW flush livers with SCS (group 2), RT LR flush livers with SCS (group 3) had lower mean AST levels at 6 h of NMP (629 ± 121 vs. 351 ± 97 units/L, p = 0.046) (Fig. 3A).

RT LR flush livers with no SCS (group 1) had the lowest mean ALT levels on NMP at 1 h (9.7 ± 4.8 units/L), 3 h (14 ± 7.0 units/L), and 6 h (25 ± 13 units/L) while cold UW flush livers with SCS (group 2) had the highest mean ALT levels at 1 h (69 ± 37 units/L) and 3 h (189 ± 63 units/L). At 6 h of NMP, group 4 livers had the highest mean ALT levels (586 ± 135 units/L). Both group 1 and group 3 livers had mean ALT levels within transplantable criteria during 6 h of NMP while group 2 and group 4 livers did not meet ALT transplantable criteria by 6 h^25,31^. The differences in mean ALT levels between group 2 and 3 livers did not reach statistical significance by 6 h (492 ± 98 vs. 278 ± 91 units/L, p = 0.069) (Fig. 3B).

RT LR flush livers with no SCS (group 1) had the lowest mean perfusate potassium levels on NMP at 1 h (5.8 ± 0.082 mmol/L) and 6 h (5.9 ± 0.096 mmol/L) while no flush livers with SCS (group 4) had the highest mean potassium levels at 3 h (7.9 ± 1.3 mmol/L) and 6 h (8.4 ± 1.0 mmol/L). There were no statistically significant differences in mean perfusate potassium levels between group 2 and 3 livers (Fig. 3C).

Finally, on gross appearance after 6 h of NMP, RT LR flush livers with no SCS (Fig. 3D) had a viable appearance devoid of infarcted lobes or cellular washout. Contrarily, cold UW flush livers (Fig. 3E) experienced significant cellular washout, giving them a pale appearance. RT LR flush livers with SCS (Fig. 3F) had a similar viable appearance to RT LR flush livers with no SCS. Finally, no flush livers (Fig. 3G) had large patchy areas of infarction and necrosis.

### Histology

Liver parenchyma histology with H&E, Reticulin, and PAS-diastase were done to assess cellular architecture and quantify the number of retained peripheral cells. TUNEL staining was used to qualitatively assess for DNA damage before and after 6 h of NMP. Before perfusion, preserved cellular architecture was seen across all groups but more DNA damage (increased TUNEL staining) was present in the no flush and the cold UW flush livers compared to the RT LR flush liver groups (Supplemental Fig. 1).

**Figure 4:**
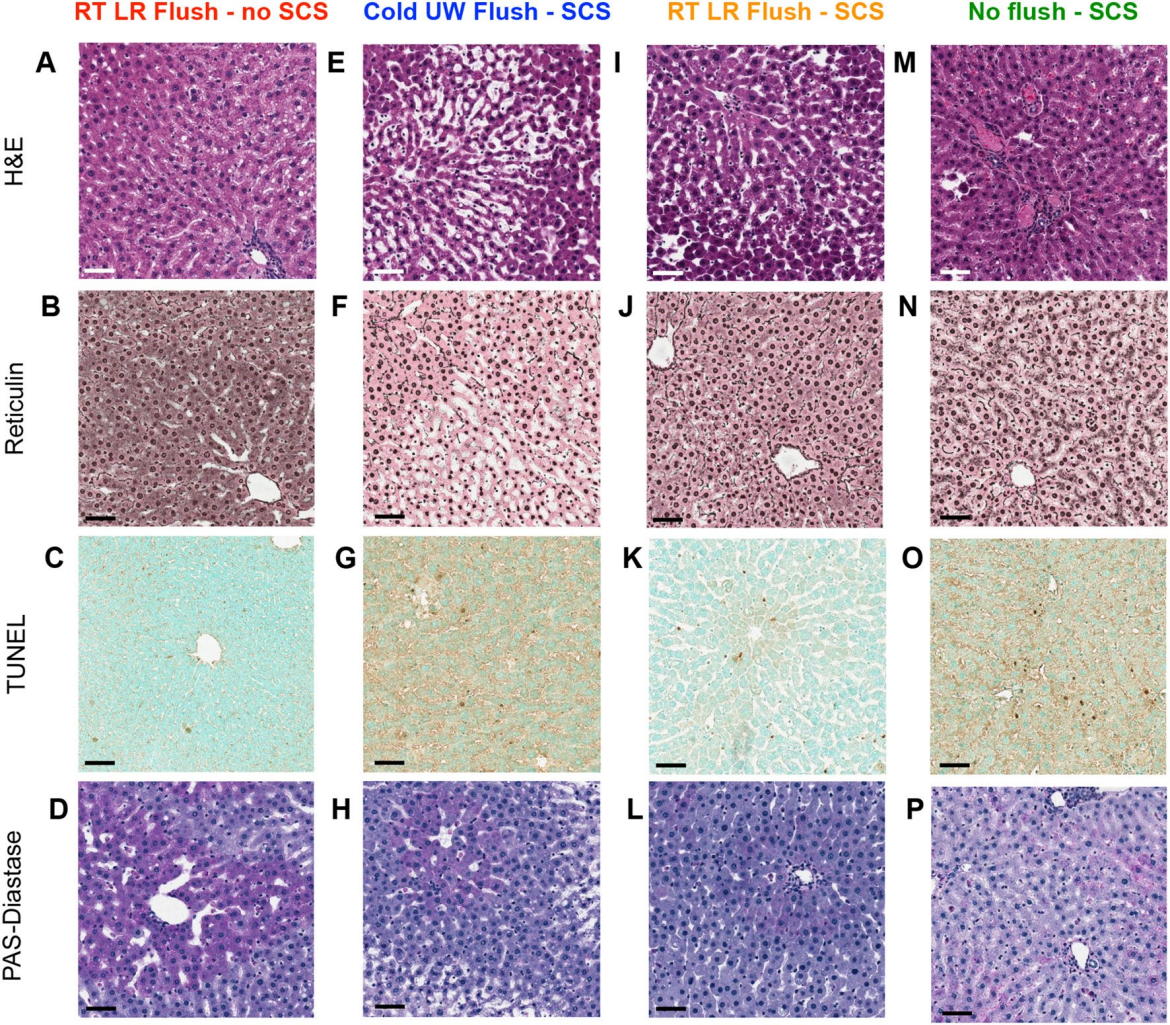
Representative histology of flush groups (in columns) after 6 h of NMP. Hematoxylin and eosin (H&E) (**A**,**E**,**I**,**M**), Reticulin (**B**,**F**,**J**,**N**), Terminal deoxynucleotidyl transferase dUTP nick end labeling (TUNEL) (**C**,**G**,**K**,**O**), and Periodic acid–Schiff (PAS)–diastase (**D**,**H**,**L**,**P**) stains. Compared to cold UW flush livers, RT LR flush livers had superior cellular architecture, less sinusoidal dilation, less DNA damage, and less retained peripheral cells. Scale bars = 100 μm.

After perfusion, group 1 livers which underwent a RT LR flush and had no SCS had preserved cellular architecture (Fig. 4A). Periportal hepatocytes however did show persistent edema in addition to marked sinusoidal dilation that is expected due to the constant, passive perfusion pressure (Fig. 4B,D). This resulted in some hepatocyte drop-out due to the widening of the subendothelial spaces. Finally, there was minimal TUNEL staining compared to the other 3 flush groups (Fig. 4C) showing focal hepatocyte apoptotic changes.

Group 2 livers which underwent a cold UW flush followed by 24 h of SCS had significant damage after perfusion which was appreciated on histology. Liver sinusoids were distended by heme pigments (due to fragmented RBCs) (Fig. 4E,F). There was exaggerated zonal differentiation with dilatation of pericentral sinusoids due to passive perfusion pressure. Periportal hepatocytes were preserved while those in pericentral area showed focal steatosis. TUNEL analysis of these livers showed that periportal liver sinusoidal endothelial cells (LSECs) had apoptotic changes with cytoplasmic staining in addition to hepatocytes with focal apoptotic changes (Fig. 4G). The sinusoids also showed remnant RBCs and lymphocytes with mostly preserved LSECs (Fig. 4H). Additional myeloperoxidase staining (Supplemental Fig. 2A) showed many fragmented granular cells with free granules in the liver sinusoids, contributing to sinusoidal distension.

Group 3 livers underwent a RT LR flush followed by 24 h of SCS and had significantly better histology compared to group 2 livers with a cold UW flush. H&E showed physiologic architecture with preserved hepatocytes and mid-zone focal steatosis but no cellular dropouts (Fig. 4I). The sinusoids were mostly clear with preserved LSECs (Fig. 4J). TUNEL analysis showed a differential distribution as hepatocytes showed good viability but apoptotic bodies were seen within intra-sinusoidal dead granular leukocytes (Supplemental Fig. 2B), and extra-sinusoidal LSECs (Fig. 4K). There were a few leukocytes and lymphocytes noted inside the sinusoids and Kupffer cells near the portal areas (Fig. 4L).

Group 4 livers underwent no flush followed by 24 h of SCS, and had the worst histology compared to the other 3 groups. Following 24 h of SCS and before perfusion, H&E showed marked congestion of the liver sinusoids with stagnant RBCs, diffuse edema, and hepatocytes exhibited periportal hydropic changes indicating early cellular degeneration (Supplemental Fig. 2C). After 6 h of NMP, H&E still showed diffuse edema and marked congestion of the liver sinusoids due to stagnant RBCs (Fig. 4M), indicating failure of NMP to remove these RPCs from the liver parenchyma. There were also focal hepatocyte emboli seen in the central veins (Supplemental Fig. 2D), indicative of hepatocyte detachment during perfusion. Reticulin stains revealed a focally disrupted reticular meshwork around the central veins (Fig. 4N). TUNEL analysis showed several intra-sinusoidal dead cells and apoptotic bodies. The overall TUNEL pattern was diffuse, patchy hepatocyte apoptotic staining (Fig. 4O). Monocytes and neutrophils were noted inside the sinusoids in the mid-zone and periportal area while lymphocytes appeared distributed within liver lobules. Kupffer cells were prominent in close proximity to portal areas (Fig. 4P).

### Correlations between retained peripheral cells and perfusion parameters of liver function/injury

We hypothesized that the type of flush technique would influence the number of RPCs in a liver graft after perfusion and possibly affect liver function during NMP. Because the flush solutions were acellular, all RPCs seen on histology were retained from procurement and not deposited during perfusion. Our results found that LR-flush livers had the lowest number of RPCs compared to UW and no flush livers. Also, the duration of SCS (LR + 24 h SCS vs. LR with no SCS) did not seem to affect the ability to flush out the retained peripheral cells during NMP (Supplemental Fig. 3).

**Figure 5:**
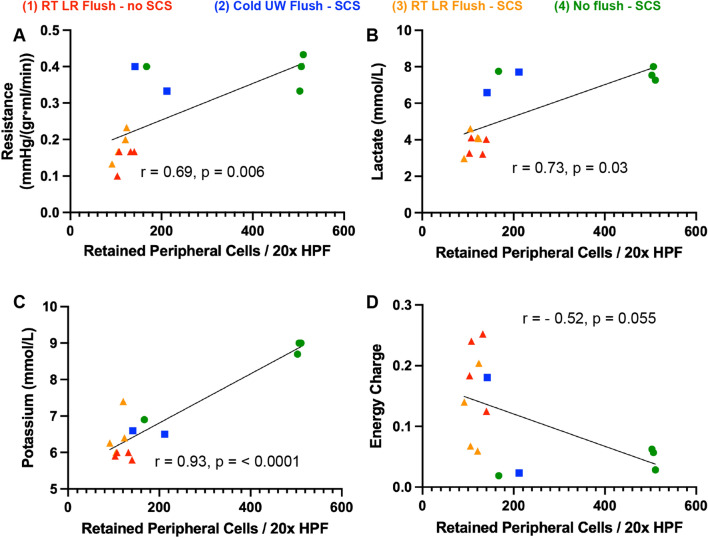
Correlations (r) between retained peripheral cells (RPCs) per 20 high powered fields (HPF) and corresponding perfusion parameters of liver function/injury. Red: RT LR flush livers with no SCS (n = 4). Blue: cold UW flush livers with SCS (n = 2). Orange: RT LR flush livers with SCS (n = 4). Green: No flush livers with SCS (n = 4). Black lines: fits of linear regression with 1,12 degrees of freedom (DFn, DFd). (**A**) Intrahepatic resistance, (**B**) lactate, (**C**) perfusate potassium, and (**D**) energy charge at 6 h of NMP versus RPCs within liver graft at 6 h of NMP. Significance level p < 0.05.

Next, resistance, lactate, EC, and potassium were correlated with the presence of RPCs per 20 HPFs on histology after 6 h of NMP using Pearson correlation coefficients (r) to measure the strength of association. Resistance (r = 0.69, p = 0.006), lactate (r = 0.73, p = 0.03), and potassium (r = 0.93, p = < 0.0001) all had significant positive correlations with the presence of RPCs in a liver allograft after 6 h of NMP, indicating that more RPCs in a liver graft were associated with higher intrahepatic resistance and cellular injury (Fig. 5A–C). EC had a negative correlation with RPCs which almost reached significance (r = − 0.52, p = 0.055), indicating that liver grafts with more RPCs had a lower metabolic energy status during perfusion (Fig. 5D). Of note, the significance of these correlations was largely driven by the no flush livers (group 4).

## Discussion

Cold ischemia in liver transplantation has detrimental effects on allograft function as a known mediator of ischemia reperfusion injury, early allograft dysfunction^4^, and primary nonfunction^5^. However, the effect of cold thermal stresses on RPCs and their impact on liver allograft performance remain understudied. Flushing solutions are responsible for the removal of RPCs at organ procurement, but the temperatures, viscosities, and compositions of these solutions play critical roles in the effectiveness of the flush. Based on the knowledge that RPCs in a liver graft and flushing solutions perform poorly in cold, we hypothesized that livers procured with a room temperature (RT) lactated ringers flush would perform better than a cold UW flush using an ex-vivo rat liver normothermic machine perfusion model and lead to less RPCs after NMP.

The effects of cold temperatures on blood cells has been extensively studied in blood transfusion medicine^32–34^ RBC morphology changes during rapid temperature decreases, which results in rouleaux formation and sludging related to cold agglutinins, cryoprecipitates, and/or cold reactive antibodies which can cause microvascular obstruction^17,35^. Similarly, platelets undergo activation-like effects when exposed to cold, which can result in platelet adhesion to the endothelium of the capillaries where tissue factor is exposed during endothelial trauma^10,36–38^. Finally, leukocytes can be activated by both cold and blood stasis, which increases cell–cell interactions, especially with the sinusoidal endothelium, leading to inflammation and cellular adhesion^39^.

This work aimed to see if these known effects of cold on blood translated to the clinical context of retained peripheral blood after flushing a liver allograft during procurement. The current clinical standard of liver procurement is a cold UW flush^40^, which results in a large temperature decrease for resident cells from 37 to 4 °C. Our results show that compared to our model of cold UW flush with NMP (group 2), livers flushed initially with RT LR (group 3) had better perfusion parameters (with respect to resistance and lactate), lower injury markers (AST/ALT), and superior histology showing less endothelial damage and TUNEL staining. A possible explanation for these findings is a more incremental temperature decrease from 37 °C in vivo, to a RT LR flush (~ 20–22 °C), and then to SCS (4 °C) which could encourage blood cell wash out and mitigate RBC, platelet, and leukocyte activation that is reflected in the lower intrahepatic resistance and lactate seen in RT LR flushed livers. Prior work has also suggested that increasing the temperature of flush solutions decreases the vascular resistance of isolated perfused organs due to dilation of capillary beds^41–43^. Prior studies in human livers have also shown that initial warm LR flush results in the least RBC retention, challenging the long-held belief of cold washout as the first step in liver procurement^19^. However, a limitation of this work is that the rate of cooling effect in rat livers with an in-situ flush is much faster compared to other animal models with larger livers. Using cold flush solutions for the purpose of metabolic depression leverages the fact that tissue deterioration slows down at decreasing temperatures^44^. Translating our results to human livers will require consideration for balancing the desired effect of warm flushes to remove peripheral cells versus the advantages of metabolic depression with cold solutions.

A second explanation for improved performance of RT LR livers compared to cold UW livers is related to flush solution composition and the resulting viscosity. Tojimbara et al. measured the viscosity of UW versus LR as initial flushing solutions and, as expected, showed that LR had lower viscosity at 4 °C (24.5 vs. 86.2 centipoise) and at 37 °C (12.2 vs. 34.7 centipoise). They subsequently showed that lower viscosity of the initial flush solution was correlated with lower vascular resistance and improved survival after rat transplant^8^. Our results were congruent with those from Tojimbara et al. in an ex-vivo NMP model, as LR (the lower viscosity solution) was more effective in removing peripheral cells from the liver allograft compared to UW. Histology comparing RT LR flush livers with cold UW flush livers after ex-vivo perfusion showed more RPCs with stagnant remnant RBCs, lymphocytes, and fragmented granular cells contributing to sinusoidal dilation in UW flush livers. While the exact mechanism of how solution composition and viscosity impact flushing effectiveness remains unknown, flushing a liver allograft with low viscosity solutions such as LR may more effectively wash out inflammatory mediators and prevent blood stagnation. Higher viscosity solutions such as UW (which contain high viscosity components such as hydroxyethyl starch), have a stronger interplay with the sphincteric action of the sinusoidal system resulting in microcirculatory disturbances, leading to hypo-perfused areas of the liver parenchyma^45^. This dynamic would help explain why AST/ALT levels were higher in UW flush livers compared to LR flush livers. The higher levels of hydroxyethyl starch (HES) in UW could also explain higher intrahepatic resistance in UW flushed livers compared to LR, as HES has been shown to have hyperaggregating effects on RBCs leading to microvascular obstruction^46,47^. Since the results of this work reveal that both temperature and solution composition (viscosity) influence the effectiveness of a flush solution, a future study is to compare RT UW to RT LR and cold UW to cold LR flushes to ascertain whether it is UW solution, flush temperature, or both that is damaging to liver allografts.

Another major aim of this study was to elucidate how RPCs during SCS damage the liver allograft. After flushing and SCS but before NMP, our histology showed that more DNA damage was present in the no flush and the cold UW flush livers compared to the RT LR flush liver groups (Supplemental Fig. 1), and these histological changes were exacerbated after 6 h of perfusion. Of note, a limitation of this analysis was that these histological changes in DNA damage were only assessed with TUNEL staining qualitatively. After NMP, unique histological and perfusion findings in no flush livers with SCS (group 4) suggest the damaging effect of RPCs is multifactorial. First, RPCs lead to congestion of the liver sinusoids, causing significant edema and obstruction of the vasculature. Even after perfusing these livers for 6 h under normothermic conditions, stagnant RPCs remained, indicating that the initial flush at time of procurement is paramount in removing peripheral blood. Subsequent poor perfusion of the liver also leads to hepatocyte drop-out, causing focal hepatocyte emboli in the central veins (seen in no flush livers). Of note, weight gain was higher in the RT LR livers w/ SCS compared to the other cold-stored liver groups but not statistically significant. The mean difference between group 3 and group 2/4 was less than 4%, which is marginal for a 11–15 g rat liver. More importantly, the differences in edema were better defined and characterized on histology which is less prone to the confounding factors compared to weight change after perfusion.

The activation of RPCs in SCS also affects hepatic performance. Free granules from activated granular leukocytes in liver sinusoids contribute to hepatic congestion. No flush livers also had the lowest energy status (ATP and EC) of all groups before and after perfusion, indicating that RPC activation affects the metabolic function of the liver on NMP. Finally, cellular death from poor flushing, seen with TUNEL staining and higher potassium levels in no flush livers, can cause cellular debris from dead cells that can disrupt the microvasculature of the liver. It is worth noting that the histological quantification of RPCs before and after perfusion within a liver graft only provides a snapshot of the intricate cellular events occurring during perfusion. Also, the significance of the correlations seen in Fig. 5 was largely driven by the no flush livers (group 4), which are not representative of standard clinical practice. Thus, future experiments using cell release assays with flowcytometry^23^ can provide real-time data on the cellular profile of the perfusate based on varying flush conditions in our ex-vivo model.

Finally, using an acellular perfusate for our NMP perfusions does not directly mimic clinical conditions which would use a blood based perfusate. The major reason the flush solutions were kept acellular was so any RPCs quantified on histology after perfusion were from the liver procurement and not reflective of any cellular deposition from a cell based perfusate during perfusion. Our group has experience with using both a pRBC and acellular based perfusates^23^, with long-term viability of rat livers after 24 h shown without an oxygen carrier^48^. Nonetheless, since clinical NMP typically uses red blood cells mixed in the perfusate, the perfusion-based viability metrics presented herein should be interpreted accordingly. Taken together, the correlations seen between the number of RPCs and perfusion parameters suggest an association between no flush livers (which had the highest number of RPCs) and poor perfusion performance compared to the other livers that all had an in-situ flush.

## Conclusions

Cold UW appears to perform worse than RT LR as an initial flush in liver procurement, indicating that a RT LR flush prior to cooling may be beneficial. Conventional wisdom suggests that the cold flush should be UW rather than LR because of its preservation properties. However, our results suggest that, during the flush, the composition and temperature of the flush is more important than the preservation properties for more complete removal of donor blood. Further studies are needed to compare RT UW to RT LR and cold LR to cold UW flushes to ascertain whether it is the composition of the UW solution or flush temperature that is primarily damaging the liver allograft. Collectively, these findings can help optimize the ideal flushing conditions for liver procurement to prevent adverse effects from retained peripheral blood.

## Supplementary Information

Supplementary Figures.

## Data Availability

The authors declare that the data supporting the findings of this study are available within the paper. Any additional data, if needed, will be provided upon request.
