# Supplementary material for: The effect of blood cells retained in rat livers during static cold storage on viability outcomes during normothermic machine perfusion
Source: Sci Rep. 2021 Nov 30;11:23128. doi: 10.1038/s41598-021-02417-6 (PMC8633375; doi:10.1038/s41598-021-02417-6)
Supplement: Supplementary file 1 — Supplementary Figures. [file 41598_2021_2417_MOESM1_ESM.docx]

**Supplemental Figure 1**. Representative histology of flush groups (in columns) before 6 hours of NMP. Hematoxylin and eosin (H&E) (**A, E, I,M**), Reticulin (**B, F, J, N**), Terminal deoxynucleotidyl transferase dUTP nick end labeling (TUNEL) (**C, G, K, O**), and Periodic acid–Schiff (PAS)–diastase (**D, H, L, P**) stains. Preserved cellular artichecture was seen across all groups but more DNA damage was seen in the no flush livers and the cold UW flush group compared to the RT LR flush livers. Scale bars = 100 μm.

**Supplemental Figure 2**. Unique histology findings. (**A**) Myeloperoxidase staining for granulocytic and monocytic cells showed free granules in liver sinusoids (asterisk). (**B**) TUNEL analysis revealed apoptotic bodies within intra-sinusoidal dead granular leukocytes (arrow) (**C**) H&E showed marked congestion of liver sinusoids with stagnant RBCs, diffuse edema, and hydropic changes indicating early cellular degeneration *before* perfusion (bracket). (**D**) Hepatocyte emboli in central vein indicative of hepatocyte detachment during perfusion (circle). Scale bar = 100 μm.

**Supplemental Figure 3.** The number of retained peripheral cells (RPCs) after 6 hour normothermic machine perfusion (NMP) based on type of flush technique. Livers flushed with room temperature LR had the fewest RPCs after perfusion compared to the other two techniques (cold UW flush and no flush).
